# Supplementary material for: Empirical methods for controlling false positives and estimating confidence in ChIP-Seq peaks
Source: BMC Bioinformatics. 2008 Dec 5;9:523. doi: 10.1186/1471-2105-9-523 (PMC2628906; doi:10.1186/1471-2105-9-523)
Supplement: Additional File 1 — A variety of html documents from the USeq web site detailing the available applications, their best usage, output file type descriptions, command line menus, etc. [file 1471-2105-9-523-S1.zip › USeqUserGuides/JavaDoc.zip!/JavaDoc/bioroot/BlastMatcher.html]

BlastMatcher


|  |  |  |
| --- | --- | --- |
| **Overview** \| **Package** \| **Class** \| **Use** \| **Tree** \| **Deprecated** \| **Index** \| **Help** | |  |
| **PREV CLASS**   **NEXT CLASS** | **FRAMES**    **NO FRAMES**     **All Classes** |  |
| SUMMARY: NESTED \| FIELD \| CONSTR \| METHOD | DETAIL: FIELD \| CONSTR \| METHOD |  |


---


## bioroot Class BlastMatcher

```
java.lang.Object
  bioroot.BlastMatcher
```

**All Implemented Interfaces:**: java.lang.Comparable

---

``` public class BlastMatcher extends java.lang.Object implements java.lang.Comparable ```

**Author:**
:   nix

---

**Constructor Summary**

`BlastMatcher(java.sql.ResultSet reagent)`
  
          Constructor passed a ResultSet containing an id, name, and sequence; watch order!

`BlastMatcher(java.lang.String oligo)`


| **Method Summary** | |
| --- | --- |
| `int` | `compareTo(java.lang.Object obj)` |
| `java.util.ArrayList` | `getBl2SeqHits()` |
| `int` | `getConcatStart()` |
| `int` | `getConcatStop()` |
| `int` | `getId()` |
| `java.lang.String` | `getName()` |
| `java.lang.String` | `getSequence()` |
| `double` | `getSortBy()` |
| `boolean` | `matches(BL2SeqHit[] hits)` |
| `void` | `setBl2SeqHits(java.util.ArrayList bl2SeqHits)` |
| `void` | `setConcatStart(int concatStart)` |
| `void` | `setConcatStop(int concatStop)` |
| `void` | `setSortBy(double sortBy)` |

**Methods inherited from class java.lang.Object**

`equals, getClass, hashCode, notify, notifyAll, toString, wait, wait, wait`

**Constructor Detail**

### BlastMatcher

```
public BlastMatcher(java.sql.ResultSet reagent)
```

:   Constructor passed a ResultSet containing an id, name, and sequence; watch order!

---


### BlastMatcher

```
public BlastMatcher(java.lang.String oligo)
```


**Method Detail**

### compareTo

```
public int compareTo(java.lang.Object obj)
```

:   **Specified by:**: `compareTo` in interface `java.lang.Comparable`

---


### matches

```
public boolean matches(BL2SeqHit[] hits)
```

---


### getId

```
public int getId()
```

---


### getName

```
public java.lang.String getName()
```

---


### getSequence

```
public java.lang.String getSequence()
```

---


### getSortBy

```
public double getSortBy()
```

---


### setSortBy

```
public void setSortBy(double sortBy)
```

---


### getBl2SeqHits

```
public java.util.ArrayList getBl2SeqHits()
```

---


### setBl2SeqHits

```
public void setBl2SeqHits(java.util.ArrayList bl2SeqHits)
```

---


### getConcatStart

```
public int getConcatStart()
```

---


### setConcatStart

```
public void setConcatStart(int concatStart)
```

---


### getConcatStop

```
public int getConcatStop()
```

---


### setConcatStop

```
public void setConcatStop(int concatStop)
```


---


|  |  |  |
| --- | --- | --- |
| **Overview** \| **Package** \| **Class** \| **Use** \| **Tree** \| **Deprecated** \| **Index** \| **Help** | |  |
| **PREV CLASS**   **NEXT CLASS** | **FRAMES**    **NO FRAMES**     **All Classes** |  |
| SUMMARY: NESTED \| FIELD \| CONSTR \| METHOD | DETAIL: FIELD \| CONSTR \| METHOD |  |


---
